# *CDX2* mutations do not account for juvenile polyposis or Peutz–Jeghers syndrome and occur infrequently in sporadic colorectal cancers

**DOI:** 10.1054/bjoc.2001.1800

**Published:** 2001-05

**Authors:** K L Woodford-Richens, S Halford, A Rowan, S Bevan, L A Aaltonen, H Wasan, D Bicknell, W F Bodmer, R S Houlston, I P M Tomlinson

**Affiliations:** Molecular and Population Genetics Laboratory, Imperial Cancer Research Fund, London WC2A 3PX, UK; Cancer and Immunogenetics Laboratory, Imperial Cancer Research Fund, Institute of Molecular Medicine, John Radcliffe Hospital, Oxford OX3 9DZ, UK; Department of Clinical Oncology, Hammersmith Hospital and Royal Postgraduate Medical School, London W12 0NN, UK; Cancer Genetics, Institute of Cancer Research, Sutton, Surrey SM2 5NG, UK; Department of Medical Genetics, Haartman Institute, University of Helsinki, Finland

**Keywords:** *CDX2*, JPS, PJS, colorectal cancer

## Abstract

Peutz–Jeghers syndrome (PJS) and juvenile polyposis (JPS) are both characterized by the presence of hamartomatous polyps and increased risk of malignancy in the gastrointestinal tract. Mutations of the *LKB1* and *SMAD4* genes have been shown recently to cause a number of PJS and JPS cases respectively, but there remains considerable uncharacterized genetic heterogeneity in these syndromes, particularly JPS. The mouse homologue of *CDX2* has been shown to give rise to a phenotype which includes hamartomatous-like polyps in the colon and is therefore a good candidate for JPS and PJS cases which are not accounted for by the *SMAD4* and *LKB1* genes. By analogy with *SMAD4CDX2* is also a candidate for somatic mutation in sporadic colorectal cancer. We have screened 37 JPS families/cases without known *SMAD4* mutations, 10 Peutz-Jeghers cases without known *LKB1* mutations and 49 sporadic colorectal cancers for mutations in *CDX2*. Although polymorphic variants and rare variants of unlikely significance were detected, no pathogenic *CDX2* mutations were found in any case of JPS or PJS, or in any of the sporadic cancers. © 2001 Cancer Research Campaign www.bjcancer.com


British Journal of Cancer (2001) 84(10), 1314–1316
© 2001 Cancer Research Campaign
doi: 10.1054/ bjoc.2001.1800, available online at http://www.idealibrary.com on IDEAL®

Short communication

### *CDX2* mutations do not account for juvenile polyposis or Peutz–Jeghers syndrome and occur infrequently in sporadic colorectal cancers

**KL Woodford-Richens[1], S Halford[2,3], A Rowan[1], S Bevan[4], LA Aaltonen[5], H Wasan[3], D Bicknell[2], WF Bodmer[2], RS Houlston[4] and IPM Tomlinson[1]**

[1]Molecular and Population Genetics Laboratory, Imperial Cancer Research Fund, London WC2A 3PX, UK; [2]Cancer and Immunogenetics Laboratory, Imperial Cancer Research Fund, Institute of Molecular Medicine, John Radcliffe Hospital, Oxford OX3 9DZ, UK; [3]Department of Clinical Oncology, Hammersmith Hospital and Royal Postgraduate Medical School, London W12 0NN, UK; [4]Cancer Genetics, Institute of Cancer Research, Sutton, Surrey SM2 5NG, UK; [5]Department of Medical Genetics, Haartman Institute, University of Helsinki, Finland

**Summary** Peutz–Jeghers syndrome (PJS) and juvenile polyposis (JPS) are both characterized by the presence of hamartomatous polyps and increased risk of malignancy in the gastrointestinal tract. Mutations of the *LKB1* and *SMAD4* genes have been shown recently to cause a number of PJS and JPS cases respectively, but there remains considerable uncharacterized genetic heterogeneity in these syndromes, particularly JPS. The mouse homologue of *CDX2* has been shown to give rise to a phenotype which includes hamartomatous-like polyps in the colon and is therefore a good candidate for JPS and PJS cases which are not accounted for by the *SMAD4* and *LKB1* genes. By analogy with *SMAD4*, *CDX2* is also a candidate for somatic mutation in sporadic colorectal cancer. We have screened 37 JPS families/cases without known *SMAD4* mutations, 10 Peutz-Jeghers cases without known *LKB1* mutations and 49 sporadic colorectal cancers for mutations in *CDX2*. Although polymorphic variants and rare variants of unlikely significance were detected, no pathogenic *CDX2* mutations were found in any case of JPS or PJS, or in any of the sporadic cancers. © 2001 Cancer Research Campaign http://www.bjcancer.com

**Keywords**: *CDX2*; JPS; PJS; colorectal cancer

Germline mutations of the *LKB1*/STK11 gene, a serine threonine kinase, on 19p13.3 have been shown to account for the majority of Peutz-Jeghers Syndrome (PJS, OMIM 175200) cases (Hemminki et al, 1998). Nevertheless, up to 50% of PJS cases have no detectable *LKB1* mutation and families unlinked to 19p13.3 have been reported (Olschwang et al, 1998). PJS patients develop hamartomas at many sites including the gastrointestinal tract, and the polyps have an arborizing structure with a smooth muscle core.

Hamartomas of the GI tract are the main characteristic of Juvenile Polyposis Syndrome (JPS, OMIM 174900), but the polyps differ from those in PJS: the juvenile polyps do not have the characteristic smooth muscle core of PJS polyps, have a greater inflammatory component, and have expanded mucin-filled cysts. Germline mutations of the *SMAD4*/DPC4 gene, a downstream regulator in the TGFβ signalling pathway, on 18q21.1, cause JPS, but can only account for about 20–25% of JPS cases (Houlston et al, 1998; Howe et al, 1998). The *SMAD4* gene has been shown to act as a tumour suppressor gene not only in JPS (Woodford-Richens et al, 2000) but also in sporadic colon and pancreatic cancers (Thiagalingam et al, 1996; Hahn, 1996 #435). Therefore, it seems likely that genes which may be considered as good candidates for the remaining PJS and JPS cases are those which are involved as part of the stepwise progression of colorectal cancer.

*Received 28 July 2000*
*Revised 22 February 2001*
*Accepted 28 February 2001*

*Correspondence to:* KL Woodford-Richens

The caudal-type homeobox gene, *CDX2* (Genbank accession numbers AF00384/5/6), encodes a transcription factor which is expressed in the intestine, and is thought to play a role in the differentiation and proliferation of intestinal epithelial cells (Drummond et al, 1997; Lorentz et al, 1997). Mice heterozygous for *CDX2* null mutations give rise to a phenotype that includes colonic polyps reported to have features of hamartomas (Chawengsaksophak et al, 1997; Tamai et al, 1999). These hamartomas do not express *CDX2*, indicating that biallelic inactivation of the *CDX2* gene is probably an important step for the growth of hamartomas (Tamai et al, 1999). *CDX2* has also been implicated in the development and progression of a subset of human colorectal cancers (da Costa et al, 1999; Mallo et al, 1997; Yagi et al, 1999).

Taken together, these findings indicate that the *CDX2* gene may be a good candidate for those cases of PJS and JPS who are not explained by mutations in *LKB1* or *SMAD4*. We have therefore screened the *CDX2* gene for germline mutations in 37 JPS patients and 10 PJS patients without known *SMAD4* or *LKB1* mutations, and for somatic mutations in 49 sporadic colorectal cancers.

### PATIENTS AND METHODS

Patients were selected who had JPS (five or more juvenile polyps or any number of juvenile polyps and a family history of JPS, plus no clinical features suggestive of other hamartoma syndromes such as Cowden, Gorlin or Bannayan-Zonana syndromes) or PJS (characteristic hamartomas of gastrointestinal tract and classical pigmentation of the lips and buccal mucosa, or with only one of these features in a familial context). Each patient had been screened previously for either *SMAD4* (JPS) or *LKB1* (PJS)

mutations, but none had been found (Hemminki et al, 1998; Houlston et al, 1998; Ylikorkala et al, 1999). Also chosen for screening were 49 colorectal cancer cell lines (C10, C32, C70, C75, C80, C84, C99, C106, C125, CAC02, COLO201, COLO205, COLO206, COLO320, COLO678, COLO741, CX1, DLD1, GP2D, GP5D, HCA46, HCA7, HCT8, HCT15, HCT116, HRA19, HT29, HT55, LIM1863, LOVO, LS174T, LS180, LS411, LS1034, PC/JW, SKC01, SW48, SW403, SW480, SW620, SW837, SW948, SW1222, SW1417, T84, VACO4A, VACO4S, VACO5, VACO10). Control samples were derived from an unselected UK population with no known cancer predisposition, but were not matched for age or sex. DNA was extracted from peripheral blood lymphocytes and cell lines using standard methods.

PCR primers for exon-by-exon amplification of *CDX2* from genomic DNA (Genbank accession numbers AF00384/5/6) were designed using Primer3 (http://www-genome.wi.mit.edu/cgi-bin/primer/primer3_www.cgi), with the forward and reverse primers both fluorescently dye-labelled (FAM, TET or HEX). PCRs were performed according to the conditions shown (Table 1), and then diluted 1:50 with distilled water. After combining the fluorescent PCR products with an internal size standard (Tamra 350, PE Applied Biosystems, Warrington, UK) and formamide, F-SSCP analysis was performed using an ABI310 sequencer (PE Applied Biosystems, Warrington, UK), under two different temperature conditions (20˚C and 35˚C). PCR products were also screened for mutations causing conformational change using the PHAST mini-gel SSCP system at 10˚C according to manufacturer's instructions, again using different temperature conditions (Pharmacia, Uppsala, Sweden).

Fragments showing both aberrant and normal migration were re-amplified using non-fluorescently labelled primers, purified using Qiaquick columns (Qiagen, Hilden, Germany) and then sequenced in both forward and reverse orientations using the ABI Big Dye Terminator kit (PE Applied Biosystems, Warrington, UK). Sequencing was performed on a subset of samples (at least 10) with normal SSCP bands to ensure all mutations were detected. No mutations were detected by direct sequencing that were not detected by F-SSCP.

### RESULTS AND DISCUSSION

No pathogenic mutations of *CDX2* were found in the 37 JPS cases or in the 49 colorectal cell lines. One of the 10 PJS cases showed aberrant migration for *CDX2* exon 3 on SSCP, which upon sequencing showed an A to T base change at nucleotide 941. This change lies in the 3′ untranslated region of the *CDX2* gene upstream of the poly A signal, and is not conserved in the mouse *CDX2* mRNA (Genbank NM007673). The mutation was not observed in any of the JPS cases, colon cancer cell lines or 100 normal control chromosomes, but its significance must remain doubtful.

A missense polymorphism was detected in exon 3, a TCT to CCT transition at nucleotide 871 which introduces a serine to proline amino acid change at codon 291. Although this might be a potential phosphorylation site, sequence analysis programs detect no evidence of homology to any consensus sequence (details not shown). Mouse *Cdx2* has a proline residue at codon 291 and is not known to be polymorphic. The frequencies of the serine and proline alleles were not significantly different in the JPS or PJS patients, in the cancer cell lines from the frequencies in a UK control cohort (Table 2) or from those frequencies previously reported in colorectal cancer (Yagi et al, 1999) (data not shown). These data therefore confirm previous suggestions that S291P is a polymorphism (Wicking et al, 1998), which is not functionally significant for JPS or PJS, although some potential functional significance as a low-penetrance cancer predisposition allele cannot entirely be excluded.

The previously reported silent polymorphism at codon 61, a CCG to CCC change (Yagi et al, 1999), was not detected in our study using SSCP, or by direct sequencing of 10 of the cancer cell line DNAs. This may result from population differences between studies. However, being a silent polymorphism the nucleotide change was anticipated not to produce any functional effect.

Thus, despite the *Cdx2* mouse knockout developing colonic hamartomas which makes *CDX2* a good candidate for JPS and PJS, we have shown that germline *CDX2* mutations do not account for the development of these two hamartoma syndromes. We have assessed linkage to the *CDX2* region (13q12.3) in the familial JPS

**Table 1** Primers and PCR conditions for *CDX2*. Exon 1 is divided into three parts so suitable fragments size for SSCP were obtained. 'Temp' indicates the annealing temperature of the PCR reaction and '$Mg^{2+}$' shows $Mg^{2+}$ concentration required

| Exon | Forward primer | Reverse primer | Temp.(˚C) | $Mg^{2+}$ (mM) |
|---|---|---|---|---|
| 1 part 1 | CAGCATGGTGAGGTCTGCT | GCGTAGCCATTCCAGTCCT | 55 | 0.5 |
| 1 part 2 | GGCAGCGAACTTGGACAG | GTTGAGCGTTTGCAGCAG | 55 | 1 |
| 1 part 3 | AGCCCCGCAGACTACCAT | CGCAGCCTCTGCTTACCTT | 55 | 0.5 |
| 2 | GCCCTCACTTCTCCTTCCTC | GTCCCCACCTGCCTCTCA | 65 | 2.5 |
| 3 | TTTTCTCCACCTTTCCATTTC | TCAGCCTGGAATTGCTCTG | 55 | 2.5 |

**Table 2** Frequencies of the polymorphic *CDX2* exon 3 alleles in JPS, PJS, colorectal cancer cell lines and a control cohort. The observed frequencies of the respective alleles did not differ significantly between patients and controls (Fisher's exact test, $P > 0.3$). The genotype frequencies do not differ significantly from Hardy–Weinberg equilibrium in any case (details not shown)

| Patients | Frequency of (t/t) homozygotes (%) | Frequency of (c/t) heterozygotes (%) | Frequency of (c/c) homozygotes (%) |
|---|---|---|---|
| Juvenile polyposis | 68 (25/37) | 30 (11/37) | 2 (1/37) |
| Peutz–Jeghers | 80 (8/10) | 20 (2/10) | 0 (0/10) |
| CRC cell lines | 72 (35/49) | 22 (11/49) | 6 (3/49) |
| Controls | 78 (40/51) | 22 (11/51) | 0 (0/51) |

© 2001 Cancer Research Campaign 

cases and found no evidence of linkage to this region, again sustaining that *CDX2* is probably not important in JPS or PJS (data not shown). The one PJS patient who possessed an A to T transversion in the 3′ UTR is unlikely to have their disease attributable to this sequence variant – unless the change contributes directly to mRNA stability or splicing in some unknown fashion. One possible way of determining the role of this variant would be to look for a 'second hit' – that is, biallelic inactivation of *CDX2* – in the hamartomas of this patient, but no such material was available. It has been suggested that the hamartomas in the knockout mouse resemble heterotopias or intercalations, that is reduplication of gut tissue in the colon, rather than true hamartomas (Beck et al, 1999). If so, it may therefore be unsurprising that no pathogenic mutations were found in two human syndromes where true hamartomas are present.

We have also shown that none of 49 colorectal cancer cell lines possesses a pathogenic mutation of *CDX2*. Previous reports have all found a low frequency of *CDX2* mutations in colon cancers (da Costa et al, 1999; Yagi et al, 1999). In one study, a single colon cancer showed biallelic inactivation of *CDX2* and restoration of expression inhibited growth (Wicking et al, 1998). Overall, it seems unlikely that somatic *CDX2* mutations are an important step in the pathogenesis of colorectal cancer. However, gene inactivation or silencing of *CDX2* via promoter methylation and/or loss of heterozygosity has not been ruled out and further investigation is required to ascertain whether this gene has an important role in the development of colorectal malignancies.

The genetic aberrations which are responsible for cases of PJS and JPS not caused by the *LKB1* and *SMAD4* genes respectively, remain elusive. Although there is debate regarding the existence of a second PJS locus, there is convincing evidence of at least one additional JPS locus. Further screening of candidate genes may be necessary to discover this gene, since the alternative – linkage analysis – will be problematic: most JPS families are too small to exclude *SMAD4* linkage and the existence of families with undetectable *SMAD4* mutations may confound the detection of critical recombinants.

### ACKNOWLEDGEMENTS

We are grateful to the Equipment Park, ICRF for help with sequencing.

### REFERENCES

Beck F, Chawengsaksophak K, Waring P, Playford R J and Furness JB (1999) Reprogramming of intestinal differentiation and intercalary regeneration in Cdx2 mutant mice. *Proc Natl Acad Sci USA* **96**: 7318–7323

Chawengsaksophak K, James R, Hammond VE, Kontgen F and Beck F (1997) Homeosis and intestinal tumours in Cdx2 mutant mice. *Nature* **386**: 84–87

da Costa L, He T, Yu J, Sparks A, Morin P, Polyak K, Laken S, Vogelstein B and Kinzler K (1999) CDX2 is mutated in a colorectal cancer with normal APC/beta-catenin signaling. *Oncogene* **18**: 5010–5014

Drummond F, Putt W, Fox M and Edwards Y (1997) Cloning and chromosome assignment of the human CDX2 gene. *Am J Hum Genet* **61**: 393–400

Hemminki A, Markie D, Tomlinson IPM, Avizienyte E, Roth S, Loukola A, Bignell G, Warren W, Järvinen H, Aminoff M, Höglund P, Pelin K, Ridanpää M, Salovaara R, Olschwang S, Bodmer WF, Olsen A, Stratton MR, de la Chapelle A and Aaltonen LA (1998) A serine/threonine kinase gene defective in Peutz–Jeghers syndrome. *Nature* **391**: 184–187

Houlston R, Bevan S, Williams A, Young J, Dunlop M, Rozen P, Eng C, Markie D, Woodford-Richens K, Rodriguez-Bigas M, Leggett B, Neale K, Phillips R, Sheridan E, Hodgson S, Iwama T, Eccles D, Bodmer W and Tomlinson I (1998) Mutations in DPC4 (SMAD4) cause juvenile polyposis syndrome, but only account for a minority of cases. *Hum Mol Genet* **7**: 1907–1912

Howe JR, Roth S, Ringold JC, Summers RW, Jarvinen H, Sistonen P, Tomlinson IPM, Houlston RS, Bevan S, Mitros FA, Stone EM and Aaltonen LA (1998) Mutations in the SMAD4/DPC4 gene in juvenile polyposis. *Science* **280**: 1086–1088

Lorentz O, Duluc I, Arcangelis AD, Simon-Assmann P, Kedinger M and Freund JN (1997) Key role of the Cdx2 homeobox gene in extracellular matrix-mediated intestinal cell differentiation. *J Cell Biol* **139**: 1553–1565

Mallo GV, Rechreche H, Frigerio JM, Rocha D, Zweibaum A, Lacasa M, Jordan BR, Dusetti NJ, Dagorn JC and Iovanna JL (1997) Molecular cloning, sequencing and expression of the mRNA encoding human Cdx1 and Cdx2 homeobox. Down-regulation of Cdx1 and Cdx2 mRNA expression during colorectal carcinogenesis. *Int J Cancer* **74**: 35–44

Olschwang S, Markie D, Seal S, Neale K, Phillips R, Cottrell S, Ellis I, Hodgson S, Zauber P, Spigelman A, Iwama T, Loff S, McKeown C, Marchese C, Sampson J, Davies S, Talbot IC, Wyke J, Thomas G, Bodmer WF, Hemminki A, Avizienyte E, de la Chapelle A, Aaltonen LA and Tomlinson IPM (1998) Peutz-Jeghers disease: most, but not all, families are compatible with linkage to 19p13.3. *J Med Genet* **35**: 42–44

Tamai Y, Nakajima R, Ishikawa T, Takaku K, Seldin M and Taketo M (1999) Colonic hamartoma development by anomalous duplication in Cdx2 knockout mice. *Cancer Res* **59**: 2965

Thiagalingam S, Lengauer C, Leach F, Schutte M, Hahn S, Overhauser J, Wilson J, Markowitz S, Hamilton S, Kern S, Kinzler K and Vogelstein B (1996) Evaluation of candidate tumor suppressor genes on chromosome 18 in colorectal cancers. *Nat Genet* **13**: 343–346

Wicking C, Simms LA, Evans T, Walsh M, Chawengsaksophak K, Beck F, Chenevix-Trench G, Young J, Jass J, Leggett B and Wainwright B (1998) CDX2, a human homologue of Drosophila caudal, is mutated in both alleles in a replication error positive colorectal cancer. *Oncogene* **17**: 657–659

Woodford-Richens K, Williamson J, Bevan S, Young J, Leggett B, Frayling I, Thway Y, Hodgson S, Kim JC, Iwama T, Novelli M, Sheer D, Poulsom R, Wright N, Houlston R and Tomlinson I (2000) Allelic loss at SMAD4 in polyps from juvenile polyposis patients and use of fluorescence in situ hybridization to demonstrate clonal origin of the epithelium. *Cancer Res* **60**: 2477–2482

Yagi O, Akiyama Y and Yuasa Y (1999) Genomic structure and alterations of homeobox gene CDX2 in colorectal carcinomas *Br J Cancer* **79**: 440–444

Ylikorkala A, Avizienyte E, Tomlinson I, Tiainen M, Roth S, Loukola A, Hemminki A, Johansson M, Sistonen P, Markie D, Neale K, Phillips R, Zauber P, Iwama T, Sampson J, Jarvinen H, Makela T and Aaltonen L (1999) Mutations and impaired function of LKB1 in familial and non-familial Peutz-Jeghers syndrome and a sporadic testicular cancer. *Hum Mol Genet* **8**: 45–51

 *© 2001 Cancer Research Campaign*

